# Refocusing the study of human–plant relations to the genus scale: Indigenous selection pressures in *Xanthosoma*

**DOI:** 10.1186/s13002-026-00919-z

**Published:** 2026-06-21

**Authors:** John Robert White, Sonia Zarrillo, Álvaro Monteros Altamirano, Marcia Peñafiel Cevallos, Roxana Tanguila, Andrea M. Cuéllar

**Affiliations:** 1 Prentice Institute for Global Population and Economy, University of Lethbridge/Iniskim, Lethbridge, Canada; 2 Prentice Institute for Global Population and Economy, University of Lethbridge/Iniskim, Lethbridge, Canada; 3 Instituto Nacional de Investigaciones Agropecuarias, Quito, Ecuador; 4 Gestión de Difusión y de Información, Instituto Nacional de Biodiversidad, Quito, Ecuador; 5 NAOQUI (Nación Originaria Quijos) and Sacha Awana, Archidona, Ecuador; 6 Prentice Institute for Global Population and Economy, Department of Anthropology, University of Lethbridge/Iniskim, Lethbridge, Canada

**Keywords:** *Xanthosoma*, Domestication, Crop wild relatives, Ethnobotany, Indigenous knowledge, Biocultural diversity, Amazonia, Human ecology, Runa

## Abstract

**Background:**

Domestication and conservation research often relies on single-species frameworks, which can obscure how Indigenous management practices applied to multiple related taxa may interact to shape shared gene pools. Studies of crops such as manioc/cassava (*Manihot esculenta*), yam (*Dioscorea* spp.), sorghum (*Sorghum* spp.), and Amazonian treegourd (*Crescentia* spp.) demonstrate that domestication frequently involves interactions among multiple cultivated and wild or semi-managed relatives. However, little is known about how these dynamics operate within the genus *Xanthosoma*, particularly in the Ecuadorian upper Amazon, a key center of diversity and domestication history for the genus. This gap limits understanding of how Indigenous management practices may influence the maintenance, erosion, or redirection of genetic diversity, with implications for crop improvement, resilience under changing environmental conditions, and the identification of underutilized plant resources.

**Methods:**

We combined ethnobotanical fieldwork in the Runa community of Mondayacu—including structured and unstructured interviews, participant observation, and genus-focused walks—with a national-level syntheses of herbarium records and ethnobotanical literature to contextualize *Xanthosoma* diversity and use across Ecuador. Interview responses were topically coded and summarized as aggregated frequencies, with patterns visualized using bar graphs. Descriptive statistics summarized reported management, while qualitative quotations and participant observation grounded interpretation.

**Results:**

Participants reported no current cultivation of *Xanthosoma sagittifolium*, despite its historical importance in Mondayacu and broader global use. In contrast, participants described a spectrum of management practices for *lalu* (*Xanthosoma purpureomaculatum*), ranging from eradication to selective retention for medicinal, zootechnical, and ritual uses. *Lalu* was the most commonly observed *Xanthosoma* species during fieldwork and was perceived as highly persistent, rapidly regrowing and spreading across forests and semi-managed areas. Decisions to remove or retain plants were trait- and context-dependent, with individuals exhibiting desirable characteristics (e.g., large, deep green leaves, minimal pest damage) preferentially spared or relocated near houses or gardens. National-level herbarium and ethnobotanical synthesis documented broader *Xanthosoma* species diversity, multifunctional uses, and possible selection pressures across Ecuadorian Amazonian Indigenous groups.

**Conclusions:**

Building on evidence from other crop systems, our findings suggest that domestication dynamics in *Xanthosoma* are not fully captured by a single-species framework. In a key center of *Xanthosoma* diversity and domestication history, differential management of multiple related species may contribute to shaping patterns of persistence and diversity within the genus. While this study does not directly measure genetic change, it identifies ethnobotanical processes that may influence the distribution and maintenance of diversity across related taxa, including those in secondary and less-studied gene pools. Approaches that account for genus-level diversity and Indigenous management practices may therefore improve understanding of domestication processes and support conservation of genetic diversity, crop improvement, and the identification of underutilized plant resources. These findings also contribute to decolonizing domestication research by highlighting how Indigenous knowledge systems and management practices shape the structure and future of crop diversity.

## Introduction

The loss of food plant diversity, including crop species and their wild relatives, poses a major threat to global food security under climate change [1, 2]. While crop wild relatives are often viewed primarily as genetic resources for industrial crop improvement [3], Indigenous and local knowledge systems demonstrate that relationships between peoples, crops, and their genetically related plants extend far beyond simple utilitarian frameworks. Indeed, as Casas, Vallejo, and Parra-Rondinel point out (4:16) “Domestication is a co-evolutionary interaction between human society and natures. numerous phenomena emerge from their interaction and are incomprehensible without this consideration.” Yet despite decades of scholars [4–6] calling for a wider analytical lens to incorporate practices such as incipient domestication and in situ management, domestication and conservation studies too often remain single species centric in biocultural theory and method. A broader framing can help reveal processes that are difficult to detect when attention is restricted to a single focal crop and the domestication dynamics affecting that species alone. Studying these interactions at wider levels, such as the landscape and genus levels, can provide a broader and more realistic basis for understanding the ecological, genetic, and cultural processes that contribute to the emergence, maintenance, and erosion of crop gene-pool diversity.

A growing body of research has demonstrated that domestication processes often extend beyond single species to involve interactions among multiple cultivated and wild or semi-managed relatives. For example, studies of manioc/cassava (*Manihot esculenta*) have shown that farmers actively incorporate wild relatives and sexual reproduction into management systems, shaping genetic diversity through ongoing hybridization and introgression [7–10]. Similar dynamics have been documented in yam (*Dioscorea* spp.) [11], sorghum (*Sorghum* spp.) [12], and Amazonian treegourd (*Crescentia* spp.) [13]. In Mesoamerica, research on columnar cacti has shown that multiple related taxa can be subject to varying degrees of management and selection within shared biocultural systems [4, 14, 15]. Together, these studies demonstrate that domestication often unfolds across species complexes and landscapes rather than discrete, isolated lineages.

Scholars have pointed to the importance of these dynamics in crops that are both sympatric and not with their genetically related relatives [16, 17]. Interactions between crops and wild relatives can also be modulated by a range of cultural practices (e.g. social shaming of farmers collecting wild yams, who may hide their use of these genetic resources from peers, obscuring their genetic inclusion in the gene pool) [18]. Such ongoing domestication of multiple related species in an area, including the reiterative use of feral populations, has been demonstrated in a range of other biocultural environments, including long lived tree species like Amazonian tree grapes (*Pourouma* spp.) [19]. Ethnobotanical syntheses further show that multiple species within the same genus are often co-managed by a single cultural group [20], underscoring the importance of examining domestication processes across related taxa rather than within single-species frameworks [4, 15].

A genus-level perspective builds on these insights by recognizing that genes can move, be maintained, and used in many ways beyond natural hybridization, including through human cultivation and propagation advances, bridge species utilization, gene insertion, and future breeding or conservation efforts that may draw on the wider shared gene pool of related species and the cultures that modify and use them [1–3]. Such research helps decolonize studies of plant genetic resources and domestication [12–15] while also supporting more informed and context-sensitive conservation strategies. The multiple biocultural genus dynamics of *Xanthosoma* species in Ecuador, provides an ideal case study for this approach.

*Xanthosoma sagittifolium* (L.) Schott, sometimes termed mandi, malanga, arrowroot, guinea yam, yautia, or tannia, is a root crop of global importance [16–21], belonging to one of the top-priority genera for gene pool conservation and a key candidate for addressing food insecurity under climate change. The crop is often consumed similarly to manioc/cassava/yuca (*Manihot esculenta*) and can serve as a staple dietary source. *Xanthasoma sagittifolium* and other *Xanthosoma* species likely experienced at least one major domestication event in Amazonia [21]. Despite its global importance, scientific research on Amazonian *Xanthosoma* remains limited compared to other crops. The upper Amazon, as a pivotal center of root crop horticulture, is crucial not only for the conservation of *Xanthosoma* genetic diversity but also for understanding how such diversity is shaped by different culturally mediated selection and land-use practices [20–24]. This region’s deep history of Indigenous horticulture underscores the importance of studying *Xanthosoma* not as a single species, but as a culturally and ecologically entangled genus. Indigenous cultural practices can shape selection dynamics, hybridization potential, and long-term persistence of crop diversity, weed, and crop wild relatives in a number of ways; these practices can include religion, resource management, teaching, social regulation, culinary preferences, craft production, and environmental ethics [2–10, 14, 15].

Despite these advances, little is known about how such multi-species domestication dynamics operate within the genus *Xanthosoma*, particularly in regions where multiple species co-occur and are differentially managed. This gap is especially significant in the Ecuadorian upper Amazon, a key center of diversity and domestication history for *Xanthosoma*, where species are widely distributed and culturally significant but have rarely been examined beyond single-species frameworks. As a result, it remains unclear how practices applied to different *Xanthosoma* species—whether cultivated, tolerated, or eradicated—may collectively shape patterns of selection, persistence, and diversity within the genus.

**Fig. 1:**
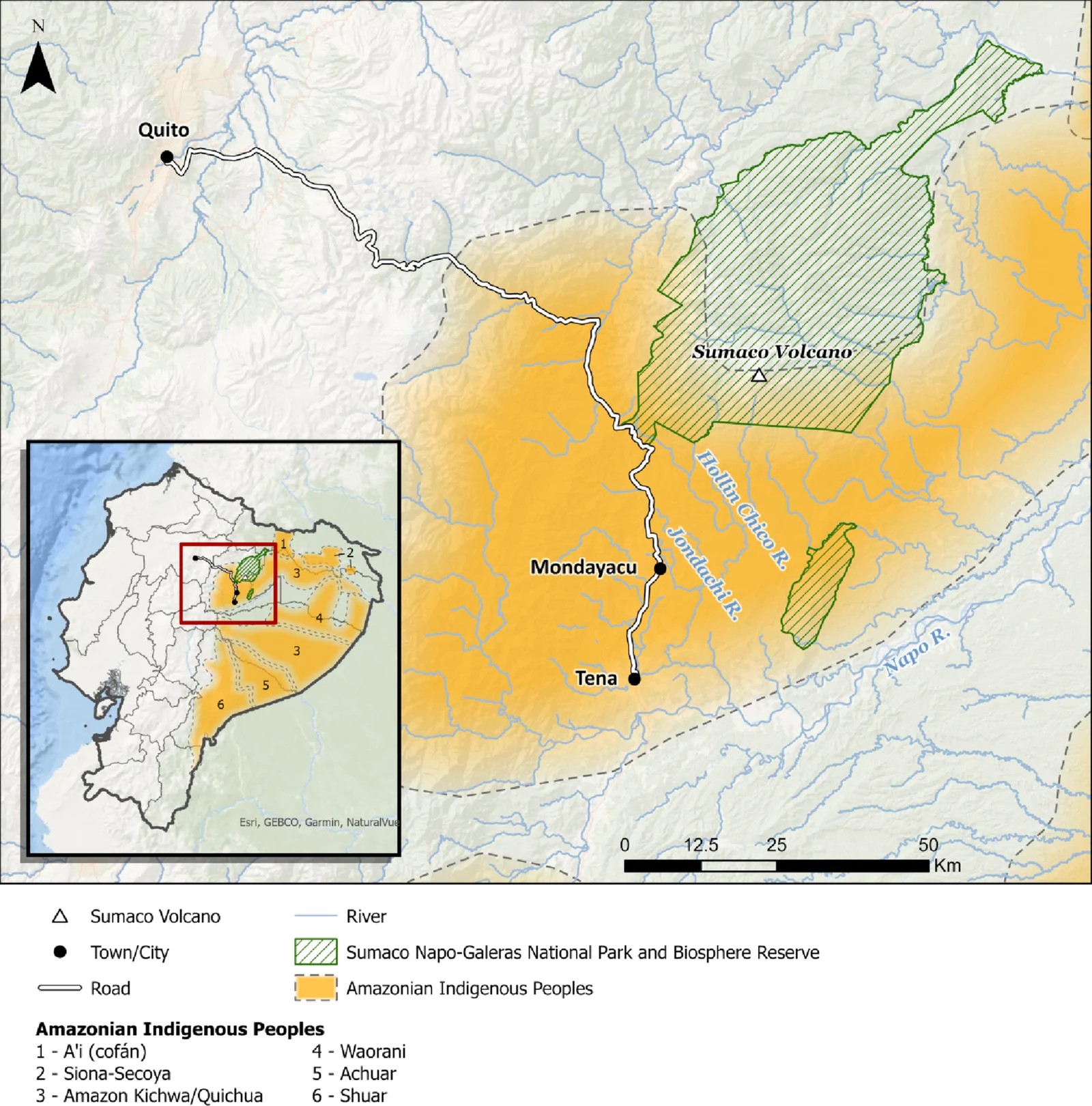
Research locations and approximate Indigenous territories (reproduced from 37). Map produced by Nick Yarmey (prentice institute for global population and economy)

To address this gap, we combined ethnobotanical fieldwork in the Runa community of Mondayacu (Fig. 1) with a national-level synthesis of herbarium and ethnobotanical records to examine how multiple *Xanthosoma* species are known, used, and managed across scales, and how these practices may shape patterns of selection and persistence within the genus [25–27], including less-studied taxa such as *Xanthosoma purpureomaculatum* (*lalu*, also sometimes termed *shikshi mandi*).

Through practices such as selective cultivation, consumption, tolerance, and weeding, different communities can exert cultural and ecological selection pressures that shape plant traits, distributions, and uses [1–6]. Such interactions blur rigid colonial boundaries between wild and cultivated and challenge fixed categories of “crop” vs. “weed” and “food” vs. “medicine,” which shift across contexts and through time, and are more accurately understood as context-dependent, relationally defined categories all potentially affecting each other through their linked (either artificially or naturally) gene pools. For the purposes of this article, we use the following definitions of crop and weed, while emphasizing that these terms are culturally dynamic and value-laden rather than fixed. A crop is understood as a plant intentionally encouraged because it is seen as useful, similarly, a weed is best defined not merely as “a plant growing where it is not wanted,” but as one judged to cause significant harm when its positive and negative attributes are weighed in a specific time and place [14]. These definitions are heuristic rather than exhaustive, and readers are referred to Ebel and Menalled [15] for a broader discussion of how edible weeds and non-crop plants are variously defined and valued across agronomy, ecology, and socio-economic contexts. Recognizing the fluidity of these classifications underscores the intertwined nature of biological and cultural diversity in domestication and beyond while catalyzing ethnobiology’s contemporary shift toward applied biocultural research, relational ecologies, and decolonial frameworks [28–33].

## Methods

### Study design and scope

“Runa” is a self-designation for many Quichua-speaking communities [34–36]. In Mondayacu and adjacent regions, Indigenous self-identification is variable and context-dependent, and may include Runa, Quichua, Quijos, or other terms. The community of Mondayacu is located in the Upper Napo region on the western edge of the Ecuadorian Amazon, approximately 20 km from the provincial capital of Tena. It overlaps part of the Sumaco Biosphere Reserve (Fig. 1) and lies within the Tropical Andean–Amazonian interface, a region recognized for its exceptional geophysical, biological, and cultural diversity and for the long-term anthropogenic contributions of Indigenous and local land-use practices [37–39].

This study integrates two complementary components: (1) ethnobotanical fieldwork conducted at the local scale in the Runa community of Mondayacu (Napo Province, Ecuador), and (2) a national-level synthesis of herbarium records and ethnobotanical literature documenting *Xanthosoma* diversity and use across Ecuador. These components are analytically distinct but combined to examine how knowledge and management of multiple *Xanthosoma* species operate across scales.

### Ethnobotanical fieldwork

Field work was conducted in the Runa community of Mondayacu between October 1, 2019, and September 30, 2022.

Our ethnobotanical approach [40] combined structured and unstructured interviews, participant observation, and field collection. Voucher specimens were analyzed at the Harvard University Herbaria and cross-referenced with biodiversity literature reviews [20] and data from published, digitized and physical herbarium collections, including the Global Biodiversity Information Facility (GBIF) [41] and the National Herbaria of Ecuador. This component supported botanical verification and helped situate local observations within broader patterns of *Xanthosoma* diversity and distribution. A snowball sampling strategy was used to identify participants knowledgeable about plant cultivation, food preparation, and traditional medicine [42–44]. After all permissions and informed consents were obtained, research was conducted with sixty adult Runa participants. Interviews and participant observations took place in a range of local contexts including Runa homes, home gardens, food production fields, and adjacent forested areas.

To reduce the risk of misidentification, we prepared high-resolution photographs and videos of known *Xanthosoma* species and used them as visual prompts during discussions. These were used alongside in situ observation during plant identification walks and structured interviews to support cross-referencing between vernacular names and botanical taxa across contexts. All structured interview figure data and their discussions in this study are based on black and white photos of a referenceable *lalu (X. purpureomaculatum)* voucher specimen collected and used in the elicitation form seen by each of the 30 participants (see Appendix 5 for an example). Although the term *lalu* refers to a vernacular category used by participants that most frequently corresponds to *Xanthosoma purpureomaculatum*, it may encompass additional morphologically similar taxa. Discussions referenced vernacular terminology used locally by participants, including distinctions between *lalu* and other named or historically recognized *Xanthosoma* taxa, rather than relying exclusively on scientific nomenclature. Voucher collections (Appendix 2) were associated with the broader field research project and regional botanical documentation rather than systematically linked to each individual unstructured interview or plant discussion event.

Of the sixty adult participants, thirty consented to further structured interviews, which were developed collaboratively with local participants and translated into locally appropriate terminology. These interviews provided more detailed data on plant use, classification, perceived changes in abundance, and management practices.

### Data analysis and presentation

**Fig. 2:**
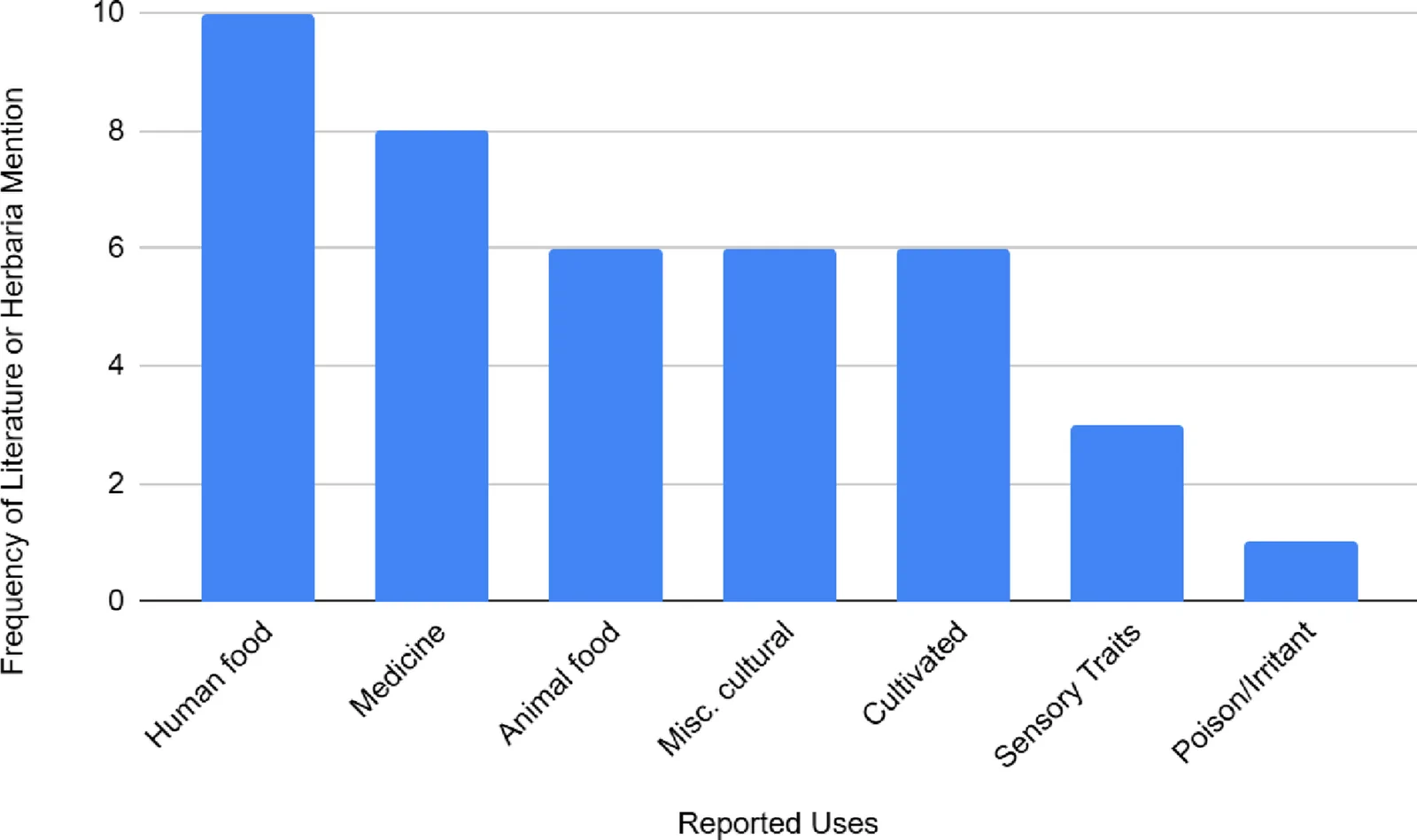
Herbaria and literature review of Xanthosoma use in Ecuador showing topical coding for many human, animal, and medicinal resources

**Fig. 3:**
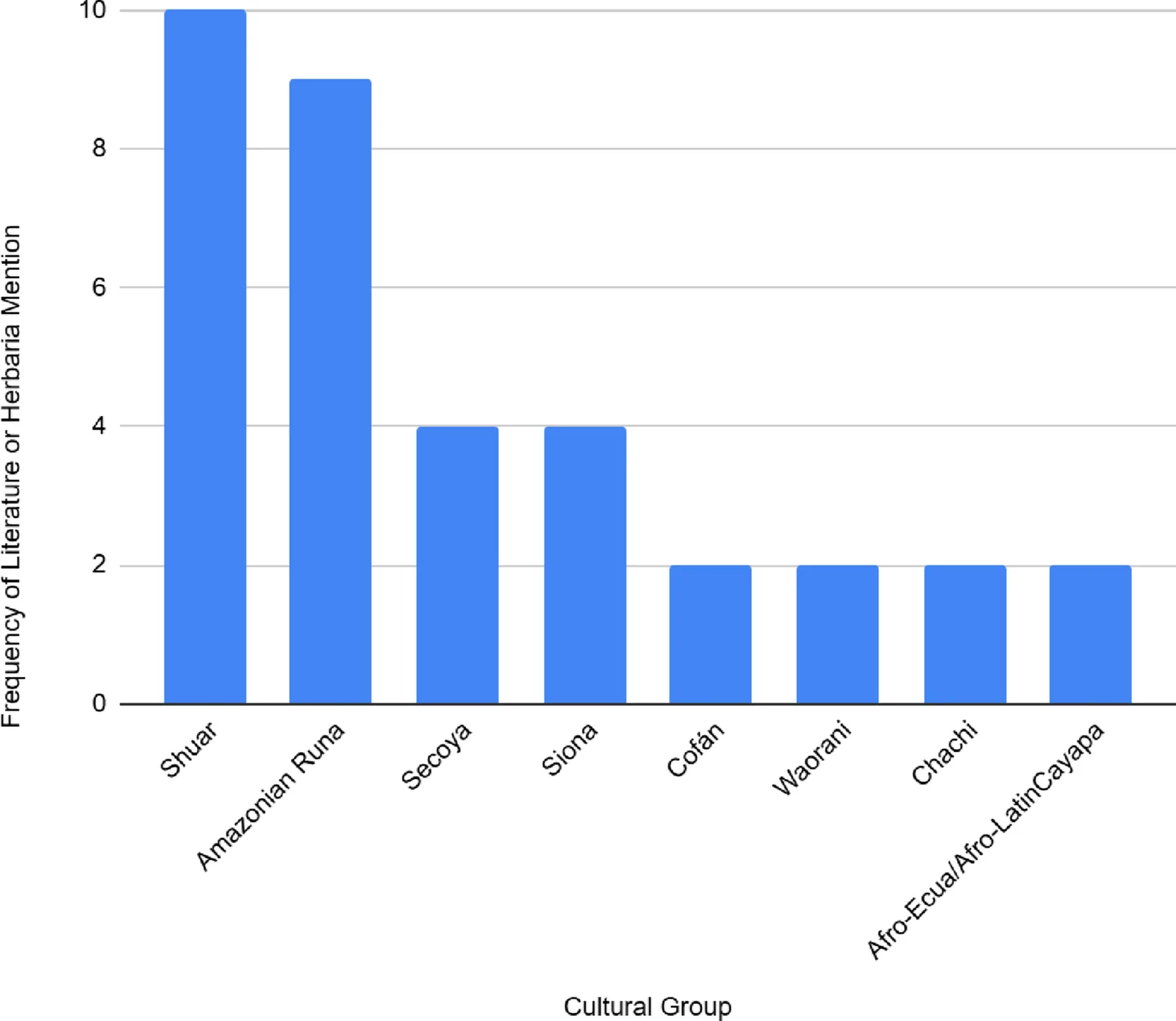
Herbaria and literature review of Ecuadorian Xanthosoma: Number of species used in diverse cultural contexts

**Fig. 4:**
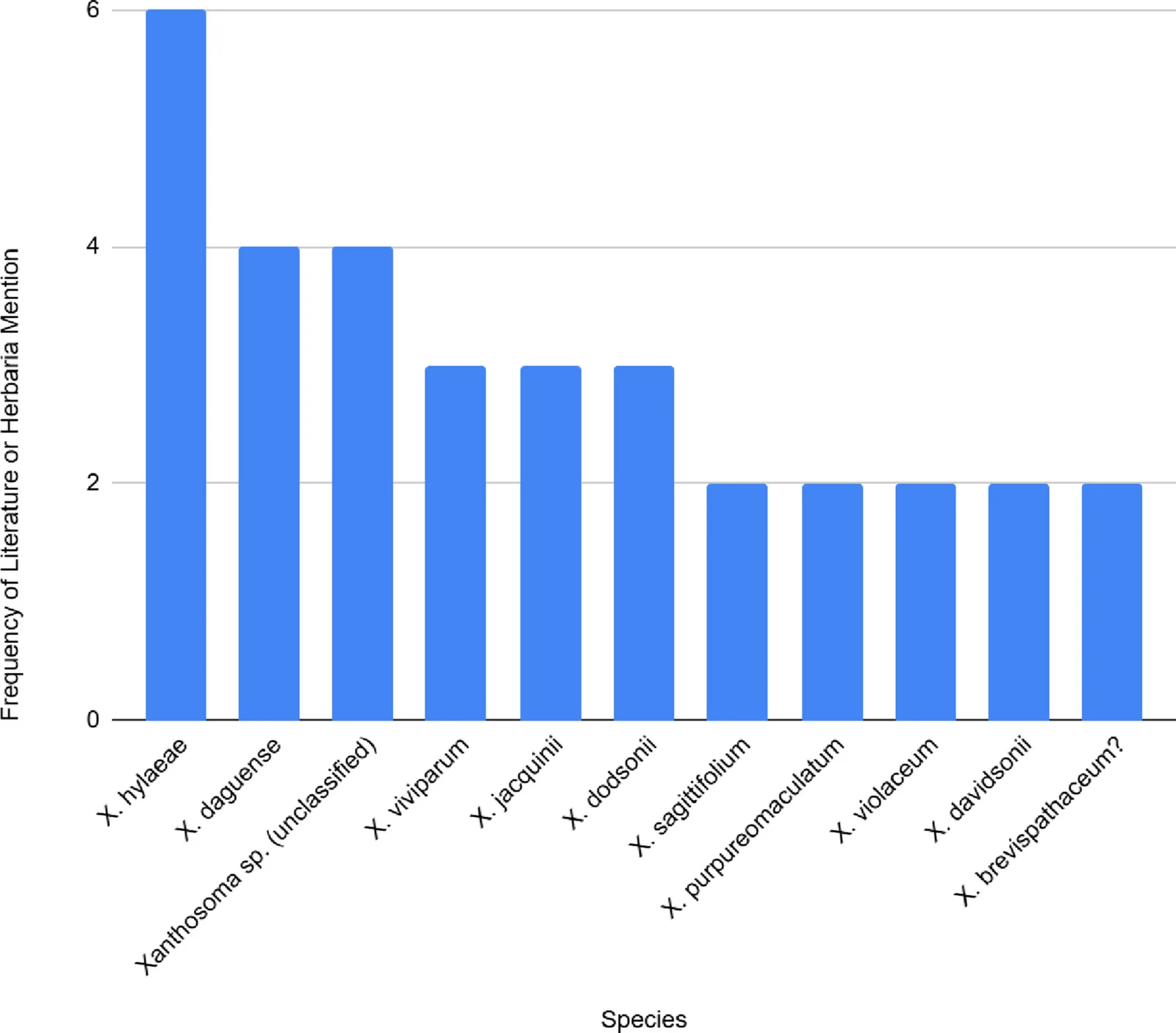
Herbaria and literature review of Ecuadorian Xanthosoma: Number of cultures using each Xanthosoma species

**Fig. 5:**
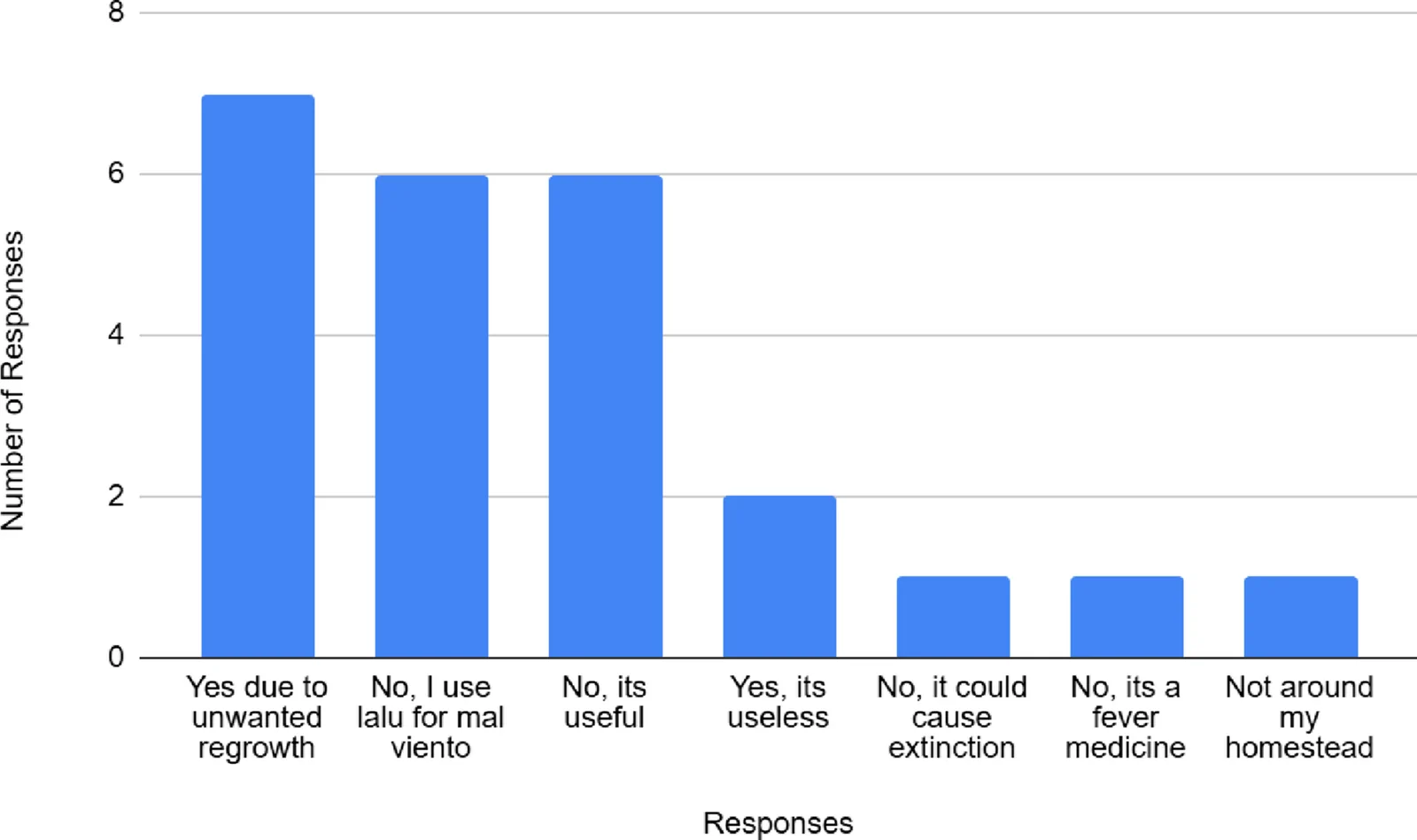
Coded multidimensional categorical responses to the question “do you weed and destroy lalu (Xanthosoma purpureomaculatum)”

**Fig. 6:**
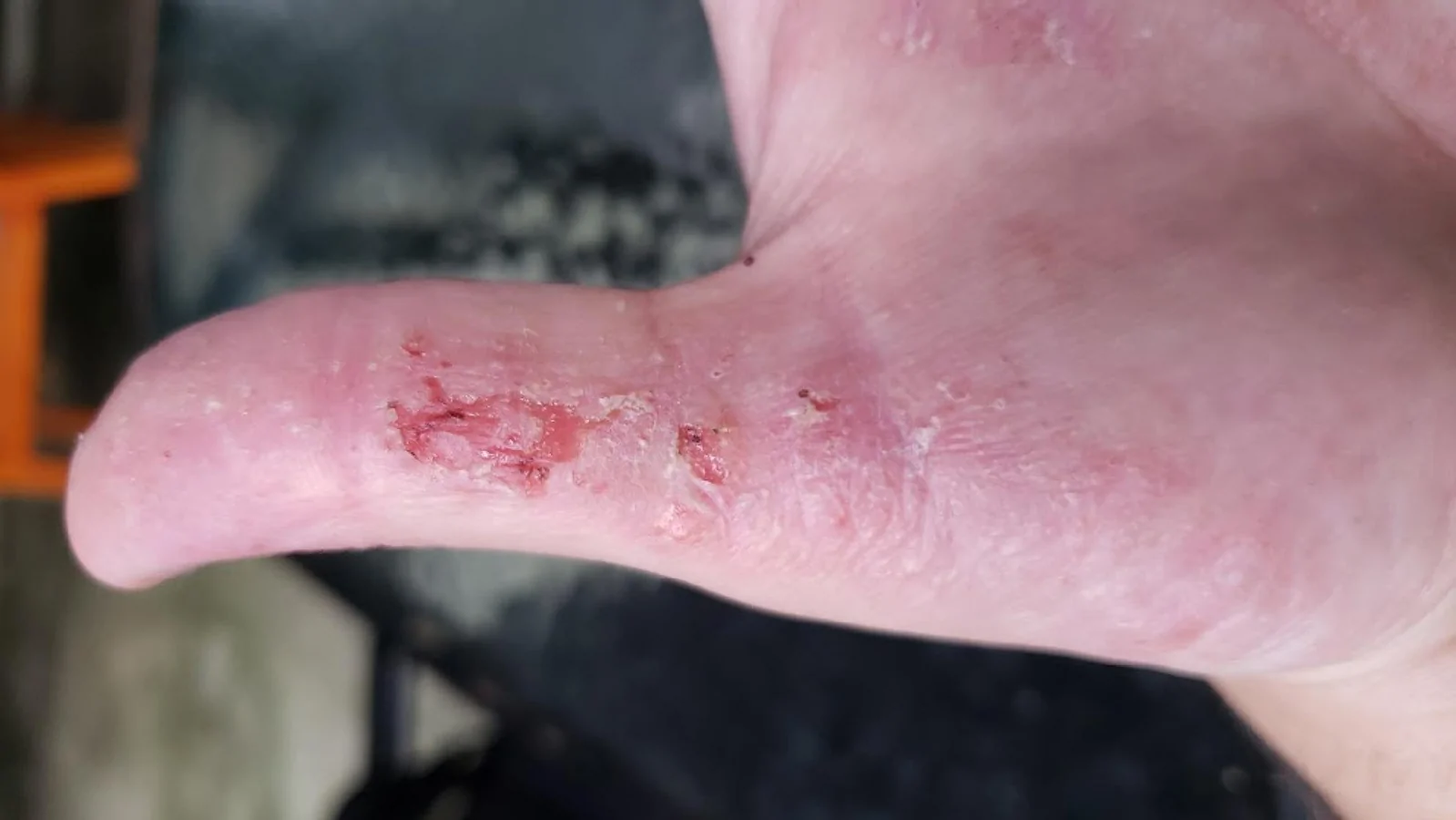
Suspected irritation from Xanthosoma purpureomaculatum on hands after voucher specimen preparation (see Appendix 4)

**Fig. 7:**
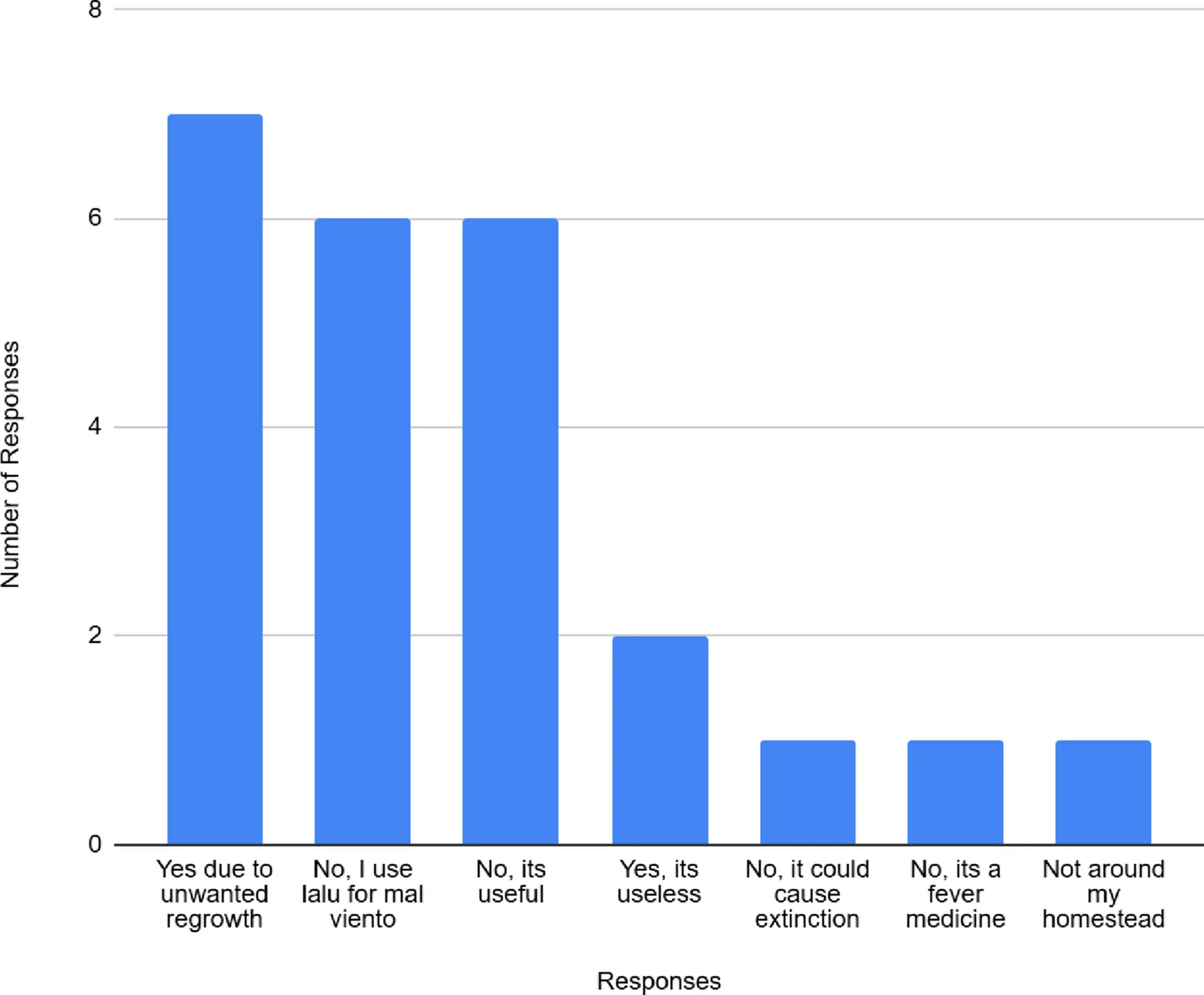
Coded multidimensional categorical responses to the question “do you weed all lalu (Xanthosoma purpureomaculatum) from garden?”

**Fig. 8:**
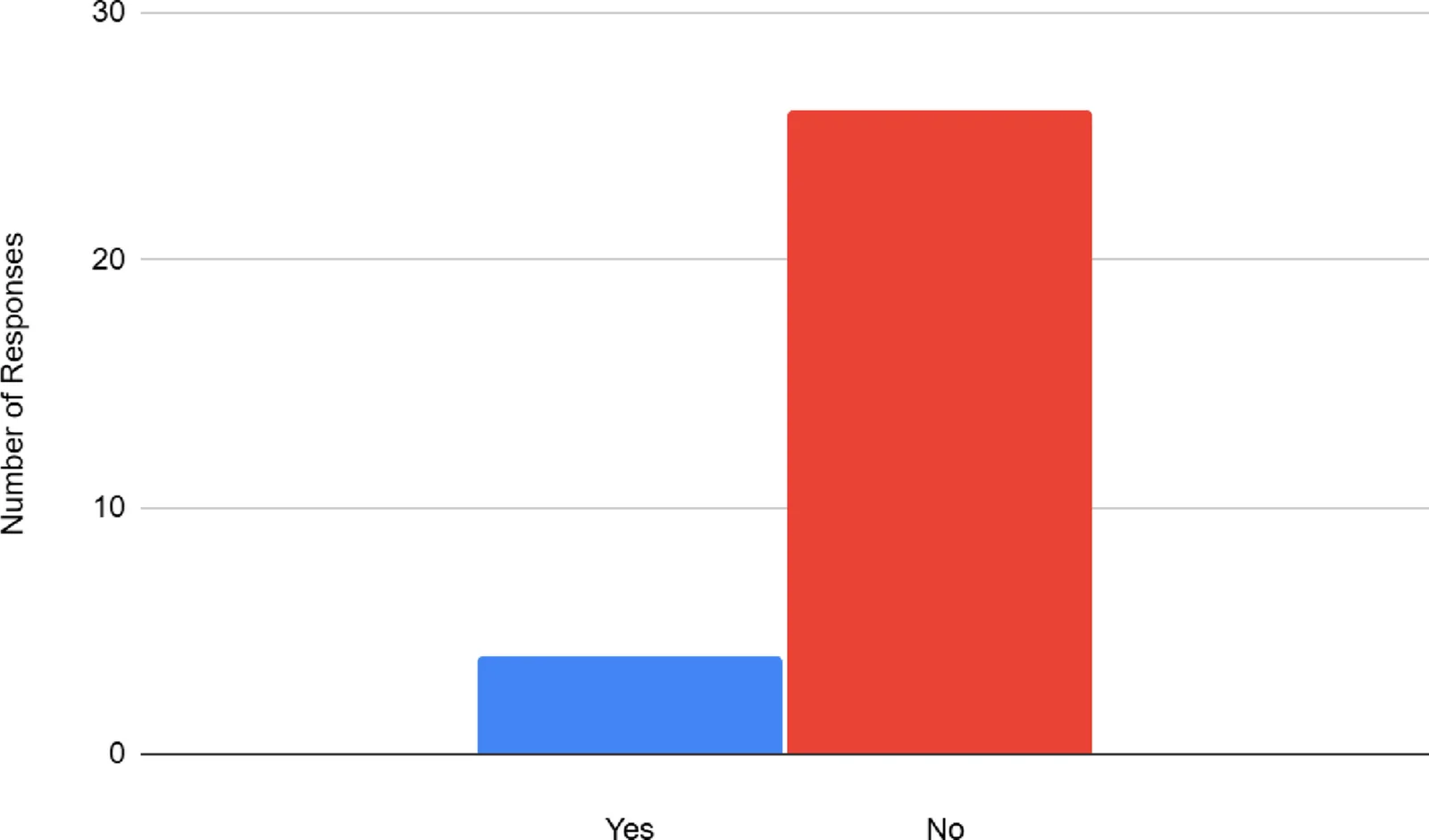
Coded interview response data: “Do you ever plant lalu (Xanthosoma purpureomaculatum)?”

Data analysis combined analytical notetaking [42–44] with qualitative coding techniques. Indexical codes were developed from initial observations, theoretical frameworks, and working hypotheses to guide data organization [44]. The coded data were subsequently transformed into thematically organized bar graphs. Each figure (Figs. 2, 3, 4, 5, 6, 7 and 8) is presented alongside key participant quotations, enabling more quantitative visualization and interpretive depth while foregrounding emic perspectives. Quotations have been anonymized for confidentiality but serialized for quantitative transparency. Neither sex nor gender were the primary analytical variables in this study but may prove useful in future studies.

To support braided knowledge production and cross-cultural comparability, vernacular plant names in Quichua/Kichwa are prioritized in the main text, with corresponding scientific identifications provided and compiled in Appendix 1. This approach is intended maintain consistency in plant identification while recognizing the variability and contextual nature of local classification systems, recognition of Indigenous knowledge, and interoperability between Indigenous and Western scientific taxonomies. Appendix 1 is provided as a reference for readers and should be understood as a starting point for comparative work rather than a definitive taxonomy. We do not wish to flatten the diversity of terms, orthographies, dialects, or languages used across communities, and the case of *lalu* illustrates the need for much more dedicated study of these classificatory relationships.

### Herbaria and literature synthesis

To contextualize our field observations beyond Mondayacu, we analyzed compiled datasets including Ecuadorian herbarium vouchers, plant occurrence records [41], and ethnobotanical literature [20] (Appendices 1–4). These sources were used to document *Xanthosoma* species diversity, reported uses, and geographic distribution across Ecuador. Figures 6, 7 and 8 summarize genus-level trends, species-level information on food, medicinal, cultural, sensory, and toxic uses, as well as the number of cultural groups reporting each species. Appendix 1 is provided as a reference and is not required to follow the main analysis.

This synthesis is not treated as a direct extension of the Mondayacu field site, but as a comparative dataset used to situate local observations [45–46] within broader patterns of *Xanthosoma* diversity and use. While the specific dynamics of each culture, species, use, valuation, and interaction necessitate dedicated research, this overview provides a basis for identifying patterns of distribution, reported uses, and potential areas for further investigation.

## Results

### Herbaria and literature results

Broad dynamics and distribution of *Xanthosoma* species knowledge was identified via herbaria and literature review among Kichwa, Shuar, Siona, Cofán, Waorani, and other cultural groups [20, 41] (Appendices 1–4). Figures 2, 3 and 4 summarize genus-level patterns, including species diversity, reported uses, and the number of cultural groups associated with each species.

Across the dataset, *Xanthosoma* species were reported in multiple use categories, including human food (10 species), animal food (6 species), medicinal applications (8 species), other cultural or cultivated purposes (6 species), sensory traits (3 species), and as poisonous or an irritant (1 species) (Fig. 2). Several species, such as *X. hylaeae*, *X. daguense*, and *X. viviparum*, are mentioned across multiple use categories, highlighting their multifunctional roles.

Multiple species were reported across different ethnolinguistic Indigenous groups. Shuar, Amazonian Runa, Secoya, and Siona communities showed the highest number of species associations (Fig. 3). At the species level, *X. hylaeae* is cited by six groups (Cofán, Secoya, Siona, Waorani, Kichwa/Quichua, and Shuar), while *X. daguense* and an unclassified *Xanthosoma* species were reported across four groups (Fig. 4). At the community level, the Shuar report the highest diversity of species (*n* = 10), followed closely by the Kichwa/Quichua (*n* = 9), with species such as *X. hylaeae*, *X. viviparum*, and *X. jacquinii* appearing consistently (Figs. 3 and 4).

### Mondayacu fieldwork results

Results from ethnobotanical fieldwork in Mondayacu are presented based on structured and unstructured interviews, incorporating participant quotations and aggregated data topically coded and visualized through bar graphs (Figs. 5, 6, 7 and 8). Research participants were asked a series of questions about how they engage with and manage *lalu* (plant images in Appendix 5), including practices of removal, retention, use, and cultivation. Unstructured inquiries were meant to reveal context tendencies along this spectrum and to understand the guiding management practices whereas structured interviews provided the following comparable quotations and coded bar graphs.

### Management of lalu: removal and retention

In response to questions about whether they remove or destroy *lalu* (Fig. 5), participants described a range of practices, including cutting, uprooting, and, in some cases, chemical treatment:

Yo elimino cortando de raíz con machete o a veces fumigo con Gramoxone y eso mata al lalu, pero con el tiempo igual vuelve a crecer.

Yes I eliminate it by cutting the root with [my]machete or sometimes fumigating with gramoxone [i.e. Paraquat, a widely used herbicide with high human and animal toxicity] and that kills *lalu*, but [shortly] it will grow back.

Male 1, 45 years of age.

Si, le saca en raíz para que no vuelva a crecer.

Yes, you take out with the roots so that it does not come back.

Male 2, 22 years of age.

Si la elimino porque es como una mala hierba que solo causa sombra.

Yes, I eliminate it because it is like a weed that only causes [unwanted] shade [for useful plants].

Female 1, 35 years of age.

Si, lo elimine porque es malo para el producto y también por que ocupa mucho espacio en la chacra, y se elimina cortando desde adentro de la raíz.

Yes, you eliminate it because it is bad for the produce and also because it occupies a lot of space on the farm, and it is eliminated by cutting from inside the root.

Male 3, 24 years of age.

### Absence of Xanthosoma sagittifolium cultivation

Participants reported no current cultivation or recent harvests of *Xanthosoma sagittifolium*, despite its historical importance in Mondayacu and its broader global use. This is despite our national scale herbaria and literature review indicating other Indigenous groups manage *X. sagittifolium* as well as multiple *Xanthosoma* species (see Fig. 2), including *lalu* (*X. purpureomaculatum*; see Appendix 1–2). Despite widespread knowledge of its former importance, participants explained that the decline in *X. sagittifolium* cultivation reflects several interacting factors. These include changes in food preferences among both adults and children, increasing crop diversity options, economic pressure to prioritize cash crops, and shrinking garden space as families grow and existing landholdings are repeatedly subdivided without new parcels being titled to households.

### Distribution and characteristics of lalu

In contrast, *lalu* was the most commonly observed *Xanthosoma* species during fieldwork and was found growing across multiple environments, including forested and semi-managed areas of Mondayacu. Its large shade-casting leaves, rapid growth, and ability to regrow from underground storage organs make it a persistent weed, according to participants. Other species of *Xanthosoma* were reported in unstructured interviews and observed in participant observation activities, but these could not be collected or botanically verified.

### Use and retention of lalu

*Lalu*, like many *Xanthosoma* species, contains irritating substances including latex, calcium oxalate needle crystals and their saponins [16] in all parts of the plant [17] that can irritate the skin (Fig. 6), prompting some Runa to eradicate entire underground systems using machetes, shovels, or digging sticks, while others reported occasional pesticide application (Fig. 5). Despite frequent removal, some participants described retaining or selectively conserving *lalu* for specific uses, particularly medicinal applications, including treatments for insect bites or “*mal aire”* (wind sickness) (Fig. 5).

Responses to questions about whether all *lalu* is removed (Fig. 7) included:

*“Cuando limpio si encuentro lalu cortamos todos*,* considero que causa hongos*,* eso me han dicho.“*.

When you weed, if you encounter *lalu*, we cut it all. I believe it causes fungi [likely plants to mold], that’s what I’ve been told.

Female 2, 38 years of age.

Si, porque estorba mi chackra, le veo como una mala hierba.

Yes, because [*lalu*] hinders my garden, I see [*lalu*] as a weed.

Male 1, 45 years of age.

Elimino la planta lalu porque es una mala hierba en la chacra y guardo algunas porque es medicina natural.

I eliminate *lalu* because it’s a weed in the garden, but I save some for natural.

medicine.

Male 4, 33 years of age.

Siempre conservo para utilizar cando coge mal aire.

I always conserve [some *lalu*] to use when someone catches *mal aire* [wind sickness].

Female 3, 46 years of age.

Cortamos algunas y otras la conservamos para cuando da mal viento esta planta es buena.

We cut some and keep some because when *mal viento* [bad wind] occurs this plant is good.

Female 4, 62 years of age.

*“Lo corto todas porque lalu vuelve a crecer*,* pero lo que crece alrededor de la casa eso si lo conservo porque sirve para limpiar mal aire. “*.

Cut all [of it] because *lalu* grows back. But what [*lalu*] grows around the house I keep because it serves to treat *mal aire* [bad air/evil air].

Female 5, 33 years of age.

### Selection, relocation, and planting practices

When asked “do you ever plant *lalu*,” (Fig. 8), the majority of respondents said that they do not. However, some participants reported retaining or relocating plants, indicating variability in practices within the community. Participants reported that only certain individuals of *lalu* were retained, typically those with very large leaves, deep green coloration, and minimal signs of pest damage, traits that participants associated with plant health or quality. These individuals were sometimes replanted near houses, gardens, or trails for accessibility.

## Discussion

In our field study, two species of *Xanthosoma* were discussed by participants, with additional species identified through herbaria vouchers and literature review [20, 41]. Participants described a range of uses and management practices, including removal, tolerance, selective retention, and relocation. These practices varied across individuals and contexts, reflecting differences in valuation and use. Some participants described *lalu* (*X. purpureomaculatum*) primarily as a weed to be eliminated, while others retained or managed specific individuals for medicinal or ritual purposes, a pattern also reported for *X. robustum* among Indigenous groups in Mexico [27]. This variability underscores that plant classification and management operate not only across cultures but also within communities.

Integrating biodiversity datasets (Figs. 2, 3, 4, 5, 6, 7 and 8) with ethnographic data enables clearer tracing of species identifications, distributions, uses, and evolutionary pressures, while revealing overlaps between Indigenous taxonomies and scientific records and help identify selection practices across a genus or within specific cultural contexts. These findings align with broader ethnobotanical research demonstrating that domestication and plant management often extend beyond single species to involve interactions among multiple cultivated and wild or semi-managed relatives [7–15, 47–55]. Rather than discrete processes bounded within individual species, domestication may operate across assemblages of related taxa shaped through diverse cultural practices and ecological interactions.

The management of *lalu* in Mondayacu illustrates how such dynamics can unfold at a fine scale. Participants selectively retained individuals exhibiting desirable characteristics, including large leaves, deep green coloration, and minimal damage, and in some cases relocated these plants closer to homes, gardens, or pathways. These practices suggest that selection operates not only through deliberate cultivation but also through differential tolerance, removal, and spatial management. The emphasis on large, deep green, minimally damaged leaves is consistent with broader ecological research linking leaf greenness, plant condition, and herbivore damage to plant performance [56–58]. At the same time, the absence of current *Xanthosoma sagittifolium* cultivation, despite its historical importance, highlights how changes in cultural preferences, land availability, and economic priorities may alter the composition of locally managed plant assemblages.

*Lalu* also plays a role in agricultural and medicinal practices. Participants described rituals associated with manioc/cassava (*Manihot esculenta*) planting. Knowledgeable women elders select vigorous *lalu* plants and translocate and maintain the population in places considered sacred or near *chagras* (cultivation plots). During planting ceremonies, *lalu* leaves are cast over manioc planting material while songs are performed, with the intention of promoting abundance, protecting crops from illness, and ensuring favorable growing conditions. In addition, *lalu* serves as a zootechnical and medicinal resource: its leaves and tubers are used as poultry feed, and its tuberous tissues and roots are applied topically to abscesses or inflammations, perhaps drawing on analgesic, antioxidant, and anti-inflammatory effects found in other species of the genus [59–61].

While participants noted both irritant (Fig. 6) and therapeutic properties of *lalu*, including soothing and treating minor insect bites, fungal infections, and stings, these observations were based on experiential knowledge rather than controlled pharmacological testing. During fieldwork, *lalu* was not observed to be ingested or processed into prepared remedies but rather used through direct contact through the application or dispersal of fresh plant material, such as beating of fresh-cut *lalu* leaves and stems that can spray the patient with its ample exudates. These practices suggest that sensory and experiential properties—such as irritation, scent, or perceived efficacy—may contribute to its continued use, although the specific mechanisms remain beyond the scope of this study. Manioc (*Manihot esculenta*) leaves are sometimes included in such a cleansing bundle and may also interact chemically with either *lalu* or the patient, and manioc leaves are commonly chewed and applied to skin ailments as a poultice.

As noted by co-author Roxana Tanguila Alvarado, drawing on community knowledge, plants locally categorized as *lalu* are not managed as singular crops or weeds, but as entities occupying shifting roles across agricultural, medicinal, and spiritual domains. Individuals are selectively removed, tolerated, or transplanted based on phenotypic traits, spatial context, and ritual function, rather than fixed taxonomic status. For example, when *lalu* or other spontaneous plants grow abundantly in areas designated for agricultural production, communities undertake maintenance activities to limit competition with cultivated crops. In contrast, in spaces not intended for cultivation (e.g. trails, peripheral areas around houses, or locations considered sacred) these plants are often preserved, provided they do not interfere with other land uses or suppress culturally or ecologically valued species. Similar relational logics have been documented in other parts of Amazonia, where plants are maintained or removed depending on their interaction with cultivated crops, their location in the landscape, and their cultural or ecological significance [7–19].

Taken together, these findings support a relational and multi-species understanding of domestication. Rather than viewing domestication as a linear process confined to individual species, it may be more accurately conceptualized as a set of interacting processes unfolding across related taxa and diverse cultural practices [4, 15]. In this study, these dynamics can be understood metaphorically as interacting ripples: actions taken with respect to one species or population—such as removal, retention, or relocation—may influence the distribution, persistence, and use of other related taxa within a shared ecological and cultural system. Interactions among related taxa may also be shaped by practices that extend beyond natural hybridization, including plant translocation, cultivation, and other forms of human-mediated movement [1–3]. This perspective is consistent with research on crop complexes and multi-species domestication systems, which demonstrate that plant evolution and management often occur across networks of related species rather than isolated lineages [9–21]. It also aligns with the gene pool framework [62–65], while extending it by emphasizing that interactions among taxa may be mediated not only by biological compatibility but also by cultural practices, spatial management, and shifting patterns of use.

Expanding analysis to the genus level may therefore provide a more comprehensive understanding of how plant diversity is maintained, transformed, or lost through human–environment interactions. In the case of *lalu*, the combined evidence from Mondayacu fieldwork and national-level synthesis (Figs. 2, 3, 4, 5, 6, 7 and 8) —including plant occurrence records [41], which were used to assess species distributions, and ethnobotanical literature [20], which was used to compile reported uses and cultural associations—suggests that multiple species are subject to distinct but overlapping forms of management, with potential implications for the persistence and distribution of genetic diversity. While this study does not directly measure gene flow or genetic change, it highlights ethnobotanical patterns that may contribute to shaping these processes over time.

An important limitation of this study concerns species identification outside of structured interviews. Although plant identification was supported through voucher collections, herbarium comparison, plant walks, and the use of photographs and videos during interviews, not all plants discussed by participants could be collected or verified taxonomically. This is especially important in *Xanthosoma*, where morphologically similar and potentially underdescribed taxa may overlap within vernacular categories such as *lalu*. Future research would benefit from systematic voucher collection linked directly to interview events, as well as genetic and morphometric analyses.

As breeding technologies evolve and cultural and research priorities shift, the boundaries and significance of primary, secondary, and tertiary gene pools are continually redefined [62–64]. These shifts are not merely classificatory but carry direct theoretical and applied consequences for how diversity is valued, conserved, and mobilized. Secondary and especially tertiary gene pools (often understudied and underrepresented in research and formal collections) constitute critical reservoirs of genetic variation for stress tolerance, disease resistance, and future crop adaptation, placing them at heightened risk of erosion, exclusion from conservation strategies, and loss of associated knowledge systems [63].

Within this context, approaches that focus narrowly on single domesticated species, presumed progenitors, dominant cultural uses, and primary/secondary gene pools, risk overlooking broader networks of hybridization, introgression, and human-mediated selection, as demonstrated in manioc/cassava, yam, sorghum, and treegourd [13, 21, 65, 66] as well as more broadly in the family Cactaceae [15, 67]. This framing obscures the role of secondary and tertiary gene pools in shaping diversity, ecological interactions, and adaptive potential under Indigenous and local management, resulting in misidentified centers of origin, underestimated gene flow, and incomplete recognition of cultural structuring processes. It also constrains theoretical understandings of domestication as an ongoing, multi-species process and limits the identification of genetic resources relevant to emerging agricultural challenges.

These limitations can also be reinforced by colonial and English-language dominance in research [68, 69], which has historically privileged particular definitions of domestication, often centered on specific industrial traits and major food crops, while marginalizing alternative domestication pathways, values, and goals held by Indigenous and local communities, including those oriented toward polyculture systems, multi-use species, and ongoing management rather than discrete endpoints.

As a result, domestication is frequently studied as a singular, goal-oriented process aligned with specific cultural frameworks, rather than as a diverse set of practices and relationships, leading to the underrepresentation of co-managed species complexes and the knowledge systems that sustain them. The historical and contemporary conflation of food, medicinal, and other use categories further distorts domestication analyses [70–72], particularly for dual-use taxa such as *Dioscorea*, where medicinal diversity remains less studied than edible lines despite shared histories of anthropogenic gene flow [73]. It is telling that research on *Dioscorea*, one of the stronger comparative cases for a genus level approach, has not yet been expanded to other yam species in Benin that may be subject to similar dynamics [11]. This gap is especially important given genus’s significance for food and medicine and the number of species under Indigenous selection or management [74]. The same concern applies to *Xanthosoma*, where attention to a small number of cultivated taxa risks obscuring the broader diversity of species, uses, and management practices that may shape domestication and conservation outcomes across the genus.

Linguistic and cultural biases also contribute, as dominant or simplified terminology can obscure co-managed species systems in written records, even where multiple taxa are recognized and embedded in Indigenous classifications [75]. Similarly, colonial or non-expert linguistic flattening—where distinct taxa (e.g., *Cucurbita*, *Lagenaria*, *Crescentia*) are grouped under generalized terms such as “calabazas—can obscure biological distinctions, species distributions, and domestication histories [75]. This flattening ignores the biocultural complexities of deep time-space [76], reinforces the marginalization of secondary and tertiary gene pools and limits both theoretical understanding and practical capacity to conserve and utilize these resources under changing environmental and socio-technical conditions.

These linguistic and cultural biases matter because they shape what forms of plant management become visible as “domestication” in the first place. More broadly, these findings contribute to ongoing efforts to reconceptualize domestication as a biocultural process that is cumulative, context-dependent, and relational. In this sense, a genus-level perspective is not only an analytical expansion, but also a decolonial one, because it draws attention to forms of Indigenous management and valuation that are often obscured by species-centered and agronomic models of domestication. It therefore changes not only the scale of analysis, but also the terms through which domestication is recognized.

## Conclusion

Building on evidence from other crop systems, our findings further suggest that domestication dynamics in *Xanthosoma* are not fully captured by a single-species framework [1–20]. In the Ecuadorian upper Amazon, a key center of diversity and domestication history for the genus, multiple *Xanthosoma* species are subject to distinct but overlapping forms of management, including removal, tolerance, selective retention, and relocation. The absence of current *Xanthosoma sagittifolium* cultivation in Mondayacu, despite its historical importance, alongside the continued management of *lalu* (*X. purpureomaculatum*), illustrates how changes in cultural practices, land use, and economic priorities may reshape which species and populations persist locally.

By extending analysis beyond a persistently species-centered model to a genus-level perspective focused on genetically related biota across time, space, and culture, this study highlights how diverse forms of plant management—including cultivated, tolerated, medicinal, and so-called weedy taxa—may generate distinct but overlapping selection pressures across multiple species within a genus. Integrating ethnobotanical fieldwork with national-level herbarium and ethnobotanical synthesis highlights how Indigenous management practices vary and may influence patterns of plant persistence, distribution, and use across related taxa. While this study does not directly measure genetic change, it identifies ethnobotanical processes that may contribute to shaping genetic diversity over time. In particular, approaches focused narrowly on single domesticated species risk overlooking the broader networks of related taxa—including those in secondary and tertiary gene pools—that may serve as critical reservoirs of genetic diversity under Indigenous management. These findings have implications for domestication research and conservation practice. Approaches that account for multi-species interactions and Indigenous management systems may improve understanding of how crop diversity is maintained, transformed, and potentially conserved within *Xanthosoma* and other crop systems with similar multi-species dynamics. They may also support efforts in crop improvement, resilience under changing environmental conditions, and the identification of underutilized plant resources.

This study also points to several directions for future research. These include systematic plant inventory surveys and transects, genetic analyses, and large-scale informatics-based reviews of ethnobotanical datasets [55], all of which would help further clarify how multi-species management practices may shape plant diversity. The genus-level lens supported here offers a complementary perspective for examining how human practices shape diversity across related taxa. In this sense, the approach is similar to a biocultural landscape perspective [4, 5, 13, 15] in its attention to relational, multi-species human–environment dynamics, but it differs in its explicit focus on shared gene pools, which may transcend any single biocultural landscape and highlight connections among genetic relatives across regions, time periods, and management contexts. At the same time, it contributes to decolonizing domestication research by challenging narrow species-centered and agronomic framings that can obscure Indigenous categories, values, and management practices. A genus-level approach therefore broadens not only what counts as domestication, but also whose knowledge, practices, and priorities are recognized as shaping the futures of crop diversity.

### Positionality statement

We write as a collaborative author team that includes both non-Indigenous and Indigenous researchers working with Indigenous peoples and on Indigenous lands, and we explicitly situate this work within the continuing realities of settler colonialism and uneven power relations in research. As authors, our training, institutional affiliations, and access to funding and publication infrastructures can unintentionally reproduce differentials in knowledge production, particularly when Indigenous knowledge is translated into academic categories, or when external timelines or institutional conventions shape what is documented and how. We therefore understand positionality not as a disclaimer, but as an ongoing responsibility: to approach collaboration with humility; to recognize partners as intellectual contributors and knowledge holders (not “informants” or “assistants”); and to remain accountable to community priorities in how knowledge is recorded, interpreted, stored, and shared. Roxana Tanguila Alvarado is an Indigenous Kichwa researcher, based in Cotundo and Archidona, who identifies as Quijos and speaks Kichwa–Shilipanu. Her contributions to this paper draw on community-based knowledge and lived experience in Quijos territory and are offered as interpretive insight to inform relational and decolonial analyses, rather than as formally elicited ethnographic or comparative data. In practice, our collaboration aims to prioritize relationship-based research, consent and local protocols, fair recognition and compensation for coresearchers where applicable, and transparent decision-making about what information is appropriate to publish versus what remains under community control. We acknowledge that no positionality statement can resolve the structural asymmetries that shape Indigenous–non-Indigenous research. Any errors, omissions, or misinterpretations in this manuscript remain our own, and we welcome correction and continued dialogue in ways that support the long-term goals of the Mondayacu community and our Indigenous collaborators.

## Data Availability

The datasets used and/or analyzed during the current study are available from the corresponding author on reasonable request.

## Appendix 1

GBIF.org (18 September 2025) GBIF Occurrence Download organized by ethnobiological label data https://doi.org/10.15468/dl.7bwded.

**Table:**

Species analysis	Details
*X. jacquinii*	w/ platanillo and bijau; camacho
*X. daguense*	Mande (two mentions)
*X. kvistii*	Medicine, leaves wrapped for swelling from the bite of the snake equis, the name is hekongochi in Afro-Latin Cayapa Esmeraldas
*X. hylaeae*	Cofan name: ata; Secoya name: cajo; Mandi; Wao name is cuentobe; Animal food according to Wao, name is kowentobe; Shuar community; Medicina, el latex desinflama la picadura de conga. Siona name is cajo; Kichwa community (multiple mentions); Achuar community
*X. purpuratum*	Mandi, mitologico, mal viento; Kichwa name, mandi, mitologico, mal vienot; Quichua community; Mitologico, mal viento, mandi
*X. helleborifolium*	Machacui mandi, medicinal, antiofidica todo; Medicine, antiofidico (infusion). Kichwa name: machacui; Kichwa community (multiple mentions)
*X. hannoniae*	In cultivated areas and forests; Shuar territory; Anise scented at anthesis (multiple mentions)
*X. sagittifolium*	Econtrada solo en alrededores; name is camacho
*X. crassilaminum*	Shuar community, food,
*X. viviparum*	Shuar community
*X. poepigii*	Scent of wintergreen
*X. atrovirens*	Miticio, se da de comer a los canes el fruto cocinado: Siona name huque
*X. diazii*	Sweet odor male spadex
*Xanthsoma sp.*	Quichua name is machacui mandi, medicinal; Weki kaho is name in Siona, edible tuber; Common names is camacho and casimbia; Tuber reportedly yellow like egg yolk, common name in Siona is pira which means papa del campa maybe, leaves eaten cut grated and cooked with fish, tuber also eaten; Camacho is common name (multiple mentions); Tuber is eaten, Secoya name is wea kaho derived from golden color of tuber, body contact reported to cause itch, in a house garden; Kofan name is tovo it is reportedly not used and is poisonous to the touch
*X. ceronii*	Cultivated plant collection
*X. reticulatum*	Mande
*X. davidsonii*	Camacho is common name
*X. perssonii*	Cultivated outside main office
*X. violaceum*	Cultivated on Galapagos chain; Shuar community, name is urianchum sanke, tuber eaten; Quichua name is ushpa mandi, tuber can be eaten cooked; Quichua name is machacui mandi, medicinal; Mandi, planted near house in central plaza
*X. dodsonii*	Camacho is common name (multiple mentions)
*X. brevispathaceum?*	Language jiv 4 shuar?; ambi sangu is name, cultivated, eaten
*X. rovae*	Cultivated outside main office
Keyword Analysis	Species
*Mandi*	*X. hylaeae*,* X. purpuratum*,* X. helleborifolium*,* X. violaceum*
*Camacho*	*X. jacquinii*,* X. sagittifolium*,* Xanthsoma sp.*,* X. davidsonii*,* X. dodsonii*
Medicinal	*X. kvistii*,* X. helleborifolium*,* Xanthsoma sp.*,* X. violaceum*
*Antiofidico*	*X. helleborifolium*,* Xanthsoma sp.*
Cultivated	*X. ceronii*,* X. perssonii*,* X. rovae*,* X. brevispathaceum?*
Anise Scented	*X. hannoniae*
Tuber Eaten	*Xanthsoma sp.*,* X. violaceum*
*Mitologico / Mal Viento*	*X. purpuratum*
Animal Food	*X. hylaeae*
Sweet Odor	*X. diazii*
Edible Tuber	*Xanthsoma sp.*
Poisonous	*Xanthsoma sp.*
Scent of Wintergreen	*X. poepigii*
Food	*X. crassilaminum*,* X. viviparum*,* Xanthsoma sp.*

**Table:**

Category counts by Xanthosoma species from GBIF data
Food (Human): 10 species
(*brasiliense*,* caracu*,* daguense*,* sagittifolium*,* crassilaminum*,* viviparum*,* violaceum*,* brevispathaceum?*,* Xanthosoma sp.*,* jacquinii* [assoc. camacho food context])
Food (Animal – vertebrates/invertebrates): 6 species
(*daguense*,* hylaeae*,* purpuratum*,* sagittifolium*,* viviparum*,* atrovirens*)
Medicinal: 8 species
(*daguense*,* helleborifolium*,* hylaeae*,* purpuratum*,* viviparum*,* kvistii*,* violaceum*,* Xanthosoma sp.*)
Misc. Cultural 6 species
(*hylaeae*,* purpuratum*,* viviparum*,* atrovirens*,* violaceum*,* Xanthosoma sp.*)
Cultivated (not necessarily food/medicine): 6 species
(*ceronii*,* perssonii*,* rovae*,* violaceum*,* brevispathaceum?*)
Sensory Traits (aroma, odor, etc.): 3 species.
(*hannoniae* – anise scent, diazii – sweet odor, *poepigii* – wintergreen scent)
Poisonous / Irritant: 1 species
(Xanthosoma sp.)

## Appendix 2

Herbaria review updated from de la Torre L, Navarrete H, Muriel P, Macía MJ, Balslev H, editors. Enciclopedia de las plantas útiles del Ecuador (con extracto de datos). Quito (Ecuador) & Aarhus (Denmark): Herbario QCA (Pontificia Universidad Católica del Ecuador) & Herbario AAU (Universidad de Aarhus); 2008. Available from: https://prolipa.com.ec/blog/wp-content/uploads/2017/08/PUB-QCA-PUCE-2008-Enciclopedia-1.pdf. (prolipa.com.ec) updated with our Xanthosoma GBIF analysis from GBIF.org (18 September 2025) GBIF Occurrence Download data https://doi.org/10.15468/dl.7bwded.

### Ecuadorian herbaria and literature review

#### In aggregate: uses of *Xanthosoma*

**Table:**

Species	Use (English)	Uso (Español)	Groups
*X. brasiliense*	Food: Corm edible	*Alimenticio: El cormo es comestible*	Zamora Chinchipe
*X. caracu*	Food: Corm edible	*Alimenticio: El cormo es comestible*	Morona Santiago
*X. daguense*	Food: Cooked corm edible	*Alimenticio: El cormo cocido es comestible*	Kichwa del Oriente – Napo
	Vertebrate food: fruit eaten by birds, tuber eaten by turtles	*Alimento de vertebrados: fruto alimento de aves*,* tubérculo alimento de tortugas*	Chachi, Afroecuatoriana, Secoya
	Medicinal: mashed leaves for snakebite	*Medicina: hojas machacadas contra mordedura de serpiente*	Chachi
	Food / ritual mentions (Mande)	—	Multiple mentions
*X. helleborifolium*	Medicinal: infusion/corm juice as antivenom for snakes & ants	*Medicina: infusión/jugo de cormo como antídoto*	Kichwa del Oriente
	Medicinal: antiofidic (snakebite)	*Medicina: antiofídico*	Multiple mentions
*X. hylaeae*	Vertebrate food: fruit eaten by mammals	Alimento de vertebrados: fruto alimento de mamíferos	Wao
	Social: ritual curses	*Social: rituales de maleficio*	Secoya
	Medicinal: latex as cicatrizant, anti-inflammatory for bullet ant sting	*Medicina: látex cicatrizante / desinflamante*	Secoya, Siona, Kichwa
	Names & use: ata (Cofán), cajo (Secoya, Siona), cuentobe/kowentobe (Wao), animal food (Wao), mandi (Kichwa, Shuar)	—	Widespread cultural
*X. purpuratum*	Vertebrate food: stem eaten by animals	*Alimento de vertebrados: tallo alimento de animales*	Kichwa del Oriente
	Invertebrate food: food for snails	*Alimento de invertebrados: alimento de caracoles*	Kichwa del Oriente, Shuar
	Social: treatment of “bad air/wind”	*Social: tratamiento del “mal aire/viento”*	Kichwa, Shuar
	Medicinal: stem scraped to stop bleeding; resin for ailments	*Medicina: tallo raspado para hemorragias*,* resina para afecciones*	Kichwa, Shuar
	Ontological associations	Ontológico, mal viento	Quichua mentions
*X. sagittifolium*	Food: cooked corm edible, leaves edible	*Alimenticio: cormo cocido*,* hojas comestibles*	Shuar, Zamora, El Oro
	Vertebrate food: corm eaten by turtles	*Alimento de vertebrados: cormo alimento de tortugas*	Chachi, Afroecuatoriana
	Common name: camacho	*—*	Multiple
*X. viviparum*	Vertebrate food: plant eaten by animals	*Alimento de vertebrados: planta alimento de animales*	Kichwa
	Social: women eat corm to gain weight	*Social: mujeres comen cormo para engordar*	Secoya
	Medicinal: latex for bullet ant sting	*Medicina: látex contra picadura de hormiga conga*	Kichwa
	Shuar community, food use noted	*—*	Shuar
*X. jacquinii*	Common name: camacho; assoc. with platanillo/bijau	*—*	Multiple
*X. kvistii*	Medicinal: leaves wrapped for swelling from snakebite (Bothrops/equis)	*Medicina: hojas envueltas para mordedura de equis*	Afro-Latin Cayapa, Esmeraldas
*X. hannoniae*	Sensory trait: anise scent at anthesis	*Aroma anisado en antesis*	Shuar
*X. crassilaminum*	Food	*Alimenticio*	Shuar
*X. poepigii*	Sensory trait: scent of wintergreen	*Aroma a gaulteria*	—
*X. atrovirens*	Social/ritual: mythic	*Miticio*	Siona
	Animal food: fruit cooked and fed to dogs	*Se da de comer a los canes el fruto cocinado*	Siona (name: huque)
*X. diazii*	Sensory trait: sweet odor in male spadix	*Olor dulce en espádice masculino*	—
*X. ceronii*	Cultivated	*Cultivada*	—
*X. reticulatum*	Food / ritual mention: Mande	—	—
*X. davidsonii*	Common name: camacho	—	—
*X. perssonii*	Cultivated (outside main office)	*Cultivada*	—
*X. violaceum*	Food: tuber eaten cooked, leaves eaten with fish	*Tubérculo comestible*,* hojas comidas con pescado*	Shuar, Quichua
	Medicinal: machacui mandi (antiofidic)	*Medicina: machacui mandi (antiofídico)*	Quichua
	Cultivated (Galapagos, house gardens)	*Cultivada*	Shuar, Quichua
	Social: mythological, mandi near central plaza	*Mitológico*,* mandi*	Quichua
*X. dodsonii*	Common name: camacho	*—*	Multiple mentions
*X. brevispathaceum?*	Food: cultivated, eaten (Shuar, Jivaroan)	*Cultivada*,* comida*	Ambi sangu (name)
*X. rovae*	Cultivated (outside main office)	*Cultivada*	—
*Xanthosoma sp.*	Food: tuber eaten, leaves grated/cooked with fish	*Tubérculo comido*,* hojas cocidas con pescado*	Siona, Secoya
	Medicinal: machacui mandi, antiofidic	*Medicina: machacui mandi*,* antiofídico*	Quichua, Siona
	Social: names camacho, casimbia, pira	*Social: nombres camacho*,* casimbia*,* pira*	Siona
	Animal/human interaction: edible tuber but body contact causes itch, sometimes poisonous	*Tóxico al tacto*	Cofán
	Cultivated in house gardens	*Cultivada*	—

#### Category counts by Xanthosoma species

- Food (Human): 10 species.
- (*brasiliense*,* caracu*,* daguense*,* sagittifolium*,* crassilaminum*,* viviparum*,* violaceum*,* brevispathaceum?*,* Xanthosoma sp.*,* jacquinii [assoc. camacho food context]*)
- Food (Animal – vertebrates/invertebrates): 6 species.
- (*daguense*,* hylaeae*,* purpuratum*,* sagittifolium*,* viviparum*,* atrovirens*)
- Medicinal: 8 species.
- (*daguense*,* helleborifolium*,* hylaeae*,* purpuratum*,* viviparum*,* kvistii*,* violaceum*,* Xanthosoma sp.*)
- Misc. Cultural 6 species.
- (*hylaeae*,* purpuratum*,* viviparum*,* atrovirens*,* violaceum*,* Xanthosoma sp.*)
- Cultivated (not necessarily food/medicine): 6 species.
- (*ceronii*,* perssonii*,* rovae*,* violaceum*,* brevispathaceum?*)
- Sensory Traits (aroma, odor, etc.): 3 species.
- (*hannoniae – anise scent*,* diazii – sweet odor*,* poepigii – wintergreen scent*)
- Poisonous / Irritant: 1 species.
- (*Xanthosoma sp.*)

**Table:**

Group	# of Xanthosoma species mentioned	Species
Shuar	10	*X. hylaeae*,* X. sagittifolium*,* X. viviparum*,* X. jacquinii*,* X. violaceum*,* X. brevispathaceum?*,* X. hannoniae*,* X. crassilaminum*,* X. purpuratum*,* X. dodsonii*
Kichwa / Quichua	9	*X. hylaeae*,* X. daguense*,* X. purpuratum*,* X. viviparum*,* X. sagittifolium*,* X. violaceum*,* X. jacquinii*,* X. kvistii*,* Xanthosoma sp.*
Secoya	4	*X. hylaeae*,* X. daguense*,* X. viviparum*,* Xanthosoma sp.*
Siona	4	*X. hylaeae*,* X. atrovirens*,* X. viviparum*,* Xanthosoma sp.*
Cofán	2	*X. hylaeae*,* Xanthosoma sp.*
Wao	2	*X. hylaeae*,* X. hylaeae*
Chachi	2	*X. daguense*,* X. daguense*
Afro-Ecuadorian/Afro-Latin Cayapa	2	*X. daguense*,* X. kvistii*

## Appendix 3

### Research, plant collection and processing methods

Research activities took place from October 1, 2019, to September 30, 2022. Our preexisting rapport with some of the Runa participants, coupled with Runa interest in this research, allowed interviews and participant observation to begin quickly. Half (n = 30) of the total participant pool (n = 60) consented to a further prolonged structured interview, locally adapted and translated with the help of Mondayacu participants, especially Janeth Grefa and Dali Angelina Alvarado Huatato. The names and places mentioned in this research are included at the express wish of those participants. We used participant observation to better contextualize our interviews by documenting how food plants, their wild relatives, and conceptualizations of and interactions with them are treated and discussed in daily life outside of an interview format. During participant observation, we looked for how interactions or discussions concerning crop wild relatives and crop diversity occurred and whether information was being conveyed that was not communicated during interviews. We also collected GPS-tagged diagnostic photographs and prepared herbarium specimens of the food plants and crop wild relatives targeted in the study. These specimens were deposited in Ecuador’s national herbaria, Ecuador’s INIAP herbaria, and Harvard University Herbaria.

Data analyses began in the field, immediately following each daily data collection period, and continued throughout fieldwork. Further data analyses and subsequent writing occurred after we departed from the field. Analytical note-taking was used for primary data analyses in this project. We created indexical codes from initial observations, theoretical frameworks, and hypotheses to code interview data, allowing for efficient retrieval and exploration of categorical data by referencing topical codes. This analytical process is also sometimes referred to as descriptive coding. Drawing on grounded theory approaches, we also created thematic codes based on participants’ responses to better explore their emic perspectives as we gained increased insight into them. Throughout the data collection process, we utilized a data interpretation and verification method similar to what is termed a constant validity check. This method assesses patterns, links, meanings, and causal explanations developed about the data through cross-examination of etic and emic explanations. We monitored for disagreement among participants, checked participants’ accuracy against more objective data to help establish primary informants, embraced evidence that might disagree with our hypotheses, attempted to make sense of negative cases through re-examination, and sought out alternative explanations. Through this recursive and reflexive examination of data, we sought to identify biases in our data collection and correct for them, both in the field and after departure.

Once a prospective specimen was targeted for collection, we took care to locate an ideal representative from the population (i.e., undamaged and complete). We then harvested the specimen, attempting to collect it intact (e.g., root system and fruits if possible), but, if needed, subdivided the specimen into several labeled parts. If the specimen was not intact, it went into its own bag so that accessory or damaged parts would not be confused with other collections. We avoided unnecessarily collecting in poor conditions when possible (e.g., extremely hot days), as these could cause specimens to wilt. For each collection, we noted the collection number, collector name, date, GPS coordinates of the collection site, and any ephemeral plant characteristics likely to fade with age (e.g., color, smell, exudates). These notations were placed on a tag tied to the plant specimen, which was left in a collecting bag or directly on the newspaper collection sheet if the specimen was to be pressed immediately. Materials used in the pressing and drying of voucher specimens included newspaper (preferably smaller than 11.5″ × 16.5″ sheets), cardboard (both standard cardboard and metal “cardboard” with uniform ventilation slits running horizontally to facilitate even heating and evaporation), blotter paper, foam padding (used to protect irregular and/or bulbous specimens and prevent them from damaging other specimens by filling in gaps to create a more homogenous layer), pens for labeling the newspaper of each voucher specimen, a plant press and compression straps, shelter, and a drying apparatus (custom ‘Iyarina Mark III’ portable tropical plant dryer).

Once a specimen was collected, it was pressed as soon as possible, as wilting can reduce the ease of identification. To press the specimen, we folded the plant in neat creases if needed to fit on the newspaper. Holes were pierced into either end of the newspaper, and the ends of specimens were tucked through them to prevent movement. Care was taken to fold plants to display their diagnostic characteristics. Some underground organs were dissected to reduce their height and display internal features. If the specimen was too large to fit on a single sheet of newspaper, we divided the specimen but labeled each sheet to indicate that they were part of a single specimen (e.g., Collection 1a, 1b, 1c). In the event of bulbous plant organs, we used herbarium foam to fill in negative spaces around the specimen. After the specimen was tucked inside a newspaper sheet with no plant material hanging over the edges, we sandwiched it between two pieces of blotter paper and two pieces of cardboard, in that order, before adding the new specimen bundle to the previously collected bundles in the plant press. After assembling the bundles, we arranged the top and bottom halves of the press so that raised panels were facing outward, avoiding damage to plant material when tightened. To tighten the plant press, we oriented the compression straps so that they were evenly spaced apart with the buckles on the top of the boards, then pulled the strap ends in opposite directions to maintain even pressure on all areas of the specimens during compression. We then applied heavy weight during compression to ensure specimens became as flat as possible in the first press, as subsequent pressings of incompletely flattened specimens that had shifted in transit could damage material.

For drying the collected and pressed specimens, JRW created a portable tropical plant dryer (Appendix 4). After assembly, we oriented the plant press horizontally and perpendicular to the heating element inside the plant press housing. We ensured that holes in the cardboard were arranged perpendicular to the heating element to allow heat and water vapor to pass through the sheets and cardboard slats, while carefully monitoring the time spent in the dryer and the rate of drying to avoid overly dried or burnt specimens. We monitored the specimens daily—and, toward the end of each plant’s drying cycle, hourly—assessing the state of dryness, swapping out newspaper and blotter paper if overly damp, and removing specimens when they were sufficiently dry and no longer cool to the touch at room temperature.

## Appendix 4

Plant Voucher Specimen Dryer (Repurposed grow tent with digitally controlled exhaust and heat lamps).

## Appendix 5

Images of Lalu (X. purpureomaculatum), including and example of

Example of the high-resolution photographs of known *Xanthosoma* species used as visual prompts during discussions, plant identification walks, and structured interviews to support cross-referencing between vernacular names and botanical taxa across contexts. Due to local technical limitations, including poor-quality colour printing, printed prompts were used in black and white. These photographs were supplemented by colour videos viewed on a tablet:
